# Motivational Effects of Methylphenidate are Associated with GABRA2 Variants Conferring Addiction Risk

**DOI:** 10.3389/fnbeh.2015.00304

**Published:** 2015-11-18

**Authors:** Theodora Duka, Claire I. Dixon, Leanne Trick, Hans S. Crombag, Sarah L. King, David N. Stephens

**Affiliations:** School of Psychology, University of Sussex, FalmerBrighton, UK

**Keywords:** psychostimulant, conditioned reinforcer, drug addiction, reward, behavioral sensitization, cocaine

## Abstract

**Background**: Variations in the GABRA2 gene, encoding α2 subunits of GABA_A_ receptors, have been associated with risk for addiction to several drugs, but the mechanisms by which variations in non-coding regions of GABRA2 increase risk for addictions are not understood. Mice with deletion of GABRA2 show deficits in the ability of psychostimulants to facilitate responding for conditioned reinforcers, offering a potential explanation.

**Methods**: We report human and mouse studies investigating a potential endophenotype underlying this association. Healthy human volunteers carrying either cocaine-addiction “risk” or “protective” GABRA2 single nucleotide polymorphism (SNPs) were tested for their subjective responses to methylphenidate, and methylphenidate’s ability to facilitate conditioned reinforcement (CRf) for visual stimuli (CS+) associated with monetary reward. In parallel, methylphenidate’s ability to facilitate responding for a visual CRf was studied in wildtype and α2 knockout (α2^−/−^) mice.

**Results**: Methylphenidate increased the number of CS+ presentations obtained by human subjects carrying protective, but not risk SNPs. In mice, methylphenidate increased responding for a CS+ in wildtype, but not α2^−/−^ mice. Human subjects carrying protective SNPs felt stimulated, aroused and restless following methylphenidate, while individuals carrying risk SNPs did not.

**Conclusion**: Human risk SNP carriers were insensitive to methylphenidate’s effects on mood or in facilitating CRf. That mice with the gene deletion were also insensitive to methylphenidate’s ability to increase responding for CRf, suggests a potential mechanism whereby low α2-subunit levels increase risk for addictions. Circuits employing GABA_A_-α2 subunit-containing receptors may protect against risk for addictions.

## Introduction

Gene variants of the GABRA2 gene, encoding α2 subunits of GABA_A_ receptors, have been associated with risk for addiction to several drugs, including alcohol (Covault et al., 2004; Edenberg et al., 2004; Enoch et al., 2006) and cocaine (Dixon et al., 2010; Enoch et al., 2010). The neurobiological mechanisms by which variations in non-coding regions of GABRA2 translate into risk for addictions are not understood, but a potential explanation comes from rodent studies showing high density of α2-containing GABA_A_ receptors in the nucleus accumbens (Pirker et al., 2000; Dixon et al., 2010), a brain area associated with reward and motivation and implicated in drug abuse and addiction (Robinson and Berridge, 1993; Robbins and Everitt, 2007). Deletion of α2-containing receptors in the mouse (α2^−/−^ mice) reduces GABAergic currents in accumbal medium spiny neurons (MSNs) by about 30% (Dixon et al., 2010), and leads to impairments in both psychomotor sensitization to cocaine (Dixon et al., 2010) and the ability of cocaine to facilitate instrumental responding for conditioned reinforcers (Dixon et al., 2010); both phenomena are hypothesized to contribute to drug abuse by facilitating incentive learning (Robinson and Berridge, 1993; Parkinson et al., 1999; Le Merrer and Stephens, 2006). Thus α2-containing receptors are both appropriately located to influence addiction-related behaviors, and manipulation of their expression influences the motivational consequences of cocaine.

Nevertheless, it is unknown whether human gene-variants conferring increased risk for addiction alter expression of α2-containing GABA_A_ receptors, and through this mechanism affect incentive learning. Accumbens activation during reward anticipation is enhanced in human adolescents carrying the GABRA2 risk G allele of rs279858 (Heitzeg et al., 2014), suggesting an effect on incentive processing consistent with reduced inhibition of accumbens MSNs, but evidence associating risk variants with changed α2 expression is inconclusive (Haughey et al., 2008). The rs299858 single nucleotide polymorphism (SNP) creates a change in the triplet codon for amino acid residue 132, but does not change the protein sequence. This and other intronic SNPs in the vicinity (including rs279871 and rs279845 studied here) may tag a region that changes the stability/expression of the mRNA and thus alter expression of the GABRA2 gene. Some support for this interpretation comes from a recent report (Lieberman et al., 2015) that GABRAA2 mRNA in neural cell cultures derived from rs279858*C allele (risk allele) carriers is lower. The question thus remains whether, and to what extent, the mouse behavioral findings provide a clue as to how the human GABRA2 genetic variations confer risk for addictive behavior.

To address this question, we carried out parallel studies in humans and mice to establish the extent to which the findings from α2^−/−^ mice inform those from humans with addiction-associated GABRA2 gene variants. Integrating data from animal and human experiments is often hindered by the use of different experimental measures in the two species (Stephens et al., 2010). In order to integrate our mouse and human experiments, we developed a human homolog of the conditioned reinforcement (CRf) measure we use in mice, allowing us to draw close parallels between data obtained from the two species. We then studied the ability of a psychostimulant drug, methylphenidate, to facilitate CRf in mice (WT and α2^−/−^) and humans (risk and protective genotypes).

## Materials and Methods

### Human Study

#### Participants

Healthy male and female volunteers (*n* = 397), aged 18–40 years, were recruited from staff and students at the University of Sussex. Buccal swabs were collected, DNA extracted, and genotypes determined commercially (KBiosciences; Hoddesdon, UK)^1^, using a proprietary fluorescence-based competitive allele-specific polymerase chain reaction (KASPar).

##### Classification of Participants According to Genotype

Genotyping of five SNPs allowed identification of subjects carrying risk or protective alleles, based on SNPs and a haplotype associated with cocaine addiction in our previous study (Dixon et al., 2010). Allocation of specific genotypes to groups is outlined on Table 1. The risk group possessed risk alleles G (rs279871) and A (rs279845) in homozygosity, but not the protective allele T (rs894269) nor the protective haplotype (rs894269:T, rs2119767:T, rs929128:G). The protective genotype group consisted of a combination of genotypes: They were homozygous for absence of the risk alleles (and carrying at least one copy of the protective SNP, or heterozygous for the risk alleles and homozygous for the protective SNP and haplotype). One of our risk SNPs, rs279871, is in 100% linkage disequilibrium with rs279858 widely reported by other groups (see “Discussion” Section). Twenty-six participants (15 female) with the risk genotype and 23 participants (14 female) with the protective genotype, aged 18–30 (mean 20.7) years, in good health, and taking no medication (with the exception of contraceptive pills), completed the following questionnaires: Alcohol Use Questionnaire (AUQ; Mehrabian and Russell, 1978), Drug Use Questionnaire (Duka et al., 2003) including smoking (cigarettes per day), the National Adult Reading Test (NART; Nelson and O’Connell, 1978) to evaluate verbal IQ, and the State-Trait anxiety scale (Spielberger et al., 1970).

**Table 1:** Participant genotypes (*Protective haplotype is rs894269 (T), rs2119767 (T), rs9291283 (G)).

SNPS/haplotypes	Risk	Protective		
rs279871	G:G	A:A	A:A	G:A
rs279845	A:A	T:T	T:T	T:A
rs894269	C:C	T:T	T:C	T:T
*Protective Haplotype	0 copies	2 copies	0/1 copy	2 copies
Methylphenidate group	*n* = 12	*n* = 3	*n* = 6	*n* = 2
Placebo Group	*n* = 14	*n* = 1	*n* = 11	*n* = 1

*The combination of genotypes selected for each SNP/haplotype are shown and number of participants for each genotype allocated to drug groups*.

Additional requirements for inclusion were that body mass index was in the range 18–28; blood pressure was <140/90 mmHg; breath alcohol concentration was 0.00 mg/l; participants were physically and mentally healthy with no alcohol or drug dependence, glaucoma, tinnitus (or family history of tinnitus); female participants were not pregnant (assessed by urine pregnancy test), or breastfeeding. Participants were required to avoid alcohol for 12 h, and recreational drugs for 5 days prior to the test session. Ethical approval was obtained from the University of Sussex ethics committee, and participants gave written informed consent. Participants received £6 per hour or course credits.

### Design

In a double-blind, placebo-controlled, between-subjects design, participants from each genotype group were randomly allocated to receive 20 mg methylphenidate or a matched placebo. Methylphenidate capsules contained 2 × 10 mg methylphenidate tablets (Medikinet^®^) inside a gelatine capsule. Placebo capsules were identical but filled with 20 mg sugar. The dose of 20 mg was chosen based on previous studies with healthy human volunteers (e.g., Elliott et al., 1997; Roehrs et al., 2004).

### Procedure

Experimental sessions lasted 5–6 h. Participants first underwent Pavlovian training, which revealed no differences between groups, before receiving the allocated treatment. To ensure participant safety, BP was measured 30 míns, and 90 mins post-drug (to coincide with peak methylphenidate plasma levels). Visual-analog scale (VAS) mood measures were assessed at 90 min. Immediately following the mood measures, the CRf test phase was carried out. At the completion of testing, BP was required to be <140/90 mmHg before participants were allowed to depart.

### Materials

#### Visual Analog Scales (VAS) of Drug Effects

A set of 9 × 100 mm VASs were labelled with adjectives related to effects of stimulant drugs (“irritable”, “stimulated”, “alert”, “restless”) intermixed with adjectives related to other drug effects (“contented”, “anxious”, “lightheaded”, “relaxed”, “tired”). The adjectives were anchored at each end with “not at all” and “very much”.

#### Profile of Mood States (POMS; Mcnair et al., 1971)

Seventy-two mood-related adjectives, rated on 5-point scales (“not at all” (0) to “extremely” (4)), provided eight factors: Anxiety, Fatigue, Depression, Anger, Vigour, Confusion, Friendliness, and Elation. For evaluating methylphenidate effects we used two composite factors: Arousal = (Anxiety + Vigour) − (Fatigue + Confusion), and Positive Mood = (Elation − Depression; de Wit and Doty, 1994). Only the two composite factors were evaluated for this study.

#### Conditioned Reinforcement Behavioral Task

**Figure 1:**
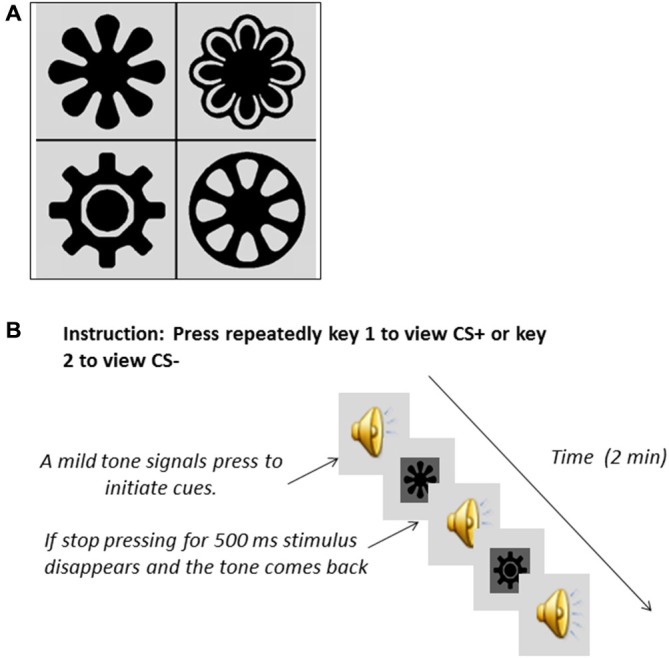
**Visual stimuli (A) used during the Pavlovian conditioning for the participants to learn associations with either monetary reward (CS+) or with absence of reward (CS−)**. Description of CRf testing **(B)**. Participants had to press repeatedly either the key associated with the CS+ or the key associated with the CS− presentation. A mild tone prompted them to start pressing if they wanted. Absence of pressing for a time window of 500 ms made the stimulus disappear.

During Pavlovian discrimination training (Austin and Duka, 2010), participants learned to associate two visual stimuli (Figure 1A), randomized across participants, with either 10p monetary reward (CS+), or no reward (CS−). The task was programmed using E-Prime v1.1 (Psychology Software Tools; Philadelphia, PA, USA) and presented on a PC. There were 50 CS+ trials and 50 CS− trials presented in blocks of 20 trials (10 CS+ and 10 CS−). At the start of the task the following instructions were presented on screen: “Each trial will begin with a fixation cross (+) in the center of the screen. Next, you will see a picture. Then you will be asked how likely you are to gain 10 pence. Rate the likelihood of gaining 10 pence on a scale of 1–9 (1 = unlikely, 5 = don’t know, 9 = likely). Press the space bar to begin”. In each trial a visual stimulus was displayed in the center of the screen for 3 s, followed in by an expectancy question which read “How likely are you to gain 10p? (1 = unlikely, 5 = don’t know, 9 = likely)”. After participants had provided their response, feedback was provided in the center of the screen for 2 s. On 90% of CS+ trials feedback consisted of the text “You gain 10p” and 10% of the time no feedback was provided. On 90% of CS− trials feedback consisted of the text “You win nothing” and 10% of the time no feedback was provided. After 20 trials participants were instructed to move 90 pence (to represent their “winnings”) from a tin located to the left of the computer keyboard into an identical tin marked “Your money tin” located to the right hand side of the computer keyboard.

During the test for CRf (Figure 1B for an illustration) responses to access the stimuli (CS+ and CS−) were recorded in the absence of monetary reinforcement. CRf was tested using two tasks, but only the task newly developed to resemble the mouse task (repeated presses CRf) is presented. The other task (press and hold CRf) was a modification of Mucha et al.’s (1998) task; no effects of drug or genotype were found and the findings are not reported here. The two versions were allocated in randomized sequence, but no effect of sequence in any of the key variables was found.

The instructions shown on screen were: “Now at the start of the trial you will hear a tone. Then you can view pictures by repeatedly pressing one of the keys marked “view”. When you stop pressing the key the picture will disappear. You can look at whichever picture you choose for as long as you choose, by pressing the appropriate key. You may switch between pictures if you wish. Press the space bar to begin”. Then followed 2 × 1 min blocks during which a neutral tone was delivered binaurally through headphones. The “Q” and “P” keys of the computer keyboard were marked clearly with the word “VIEW”, and upon pressing one of these keys at a rate of at least one press per 500 ms, the tone ceased, and CS+ or CS− was displayed in the center of the screen. If participants did not respond within a window of 500 ms, the stimulus disappeared. CS+ and CS− were allocated to the “Q” or “P” key in counterbalanced order across participants. Time spent viewing each of the stimuli was recorded. In addition, the number of times each stimulus was viewed was recorded.

During the CRf measurement participants could alternate between two keys, but either stimulus was kept in view by repeated presses on the designated key, providing a variable “duration of CS presentation”. If there were no presses within 500 ms the stimulus disappeared until reinstated by a further press, providing a variable “number of CS presentations”. Thus, whereas duration of CS presentation was the measure of conditioned incentive value expressed via capture of attention, the number of CS presentations indicates motivation to reinstate the salient stimulus.

### Mouse Study

*Animals*. Wildtype (WT; *n* = 12) and knockout (α2^−/−^; *n* = 12) male mice were bred from heterozygous pairings and maintained on a mixed 50% C57BL/6J / 50% 129SvEv background (Dixon et al., 2008). Animals were housed under a 12 h light/dark cycle (lights on at 7.00 AM), with controlled temperature (≈ 21°C) and humidity (≈ 50%). Water was available *ad libitum* within the homecage, but food was restricted to maintain body weight at approximately 85% of free-feeding weight. All experiments were carried out under the authority of the UK Animal (Scientific Procedures) Act, 1986, following institutional ethical approval.

*Drugs*. Methylphenidate hydrochloride (Sigma, UK) was dissolved in 0.9% w/v saline and administered i.p. at a volume of 10 ml/kg.

#### Methylphenidate-potentiated Conditioned Reinforcement

##### Pavlovian Conditioning

To test the ability of the α2 KO mice to form a Pavlovian association, food-deprived WT and KO mice (*n* = 12) were trained in standard conditioning chambers (MedAssociates, St. Albans, VT, USA) to retrieve sweetened food pellets (20 mg; Noyes precision pellets, Formula P, Research Diets, Inc., New Brunswick, NJ, USA), for 11 daily sessions. During each 60 min session, a 10 s (conditioned) stimulus was paired with food pellet delivery (CS+), commencing 5 s before food pellet delivery. A second stimulus (CS−) was also presented for 10 s with no food delivered. Sixteen of each CS were presented in a randomized order on a variable time schedule of 100 s. The conditioned stimuli consisted of two flashing stimulus lights (1 Hz) or a constant tone (2.9 kHz), counterbalanced between CS+ and CS−. The stimulus lights were located above and to either side of the food magazine, with the tone generator directly above the magazine. The percentage of nose pokes into the food magazine during the CS+ and CS− were recorded as a measure of discriminated approach.

*Conditioned Reinforcement*. Following training, two nosepoke apertures were introduced into the chambers, located in the wall opposite the food magazine. During a 60 min session, nosepokes into one aperture (counterbalanced) resulted in illumination of the CS+ for 1 s whilst responses into the alternative aperture resulted in a 1 s presentation of the CS−. No food reinforcers were delivered during the CRf session. The ability of the cues to act as conditioned reinforcers was assessed as higher rates of responding in the CS+ associated aperture compared to the CS− associated aperture. Animals that did not reach criterion for CRf (>75% CS+ nosepoke responses) were excluded from drug testing.

*Methylphenidate-potentiated CRf*. Mice that had acquired CRf (WT (*n* = 11); α2^−/−^ (*n* = 8)) were injected at 2-day intervals with methylphenidate (0, 0.3, 1.0 and 3.0 mg/kg) using a counterbalanced design, and tested for CRf immediately following drug administration. Drug-free Pavlovian conditioning (retraining) sessions were conducted on the days between test sessions.

#### Methylphenidate Locomotor dose Response

*Apparatus*. To assess genotype differences in the stimulant effects of methylphenidate, locomotor activity was assessed using 16 black Perspex, circular runways (internal diameter, 11 cm; external diameter, 25 cm; height, 25 cm), as described previously (Dixon et al., 2010).

*Methylphenidate locomotor activation*. WT and α2^−/−^ mice (*n* = 12 per group) were habituated to the locomotor runways for 60 min on two consecutive days. Subsequently, they were injected with methylphenidate (0, 0.3, 1.0 and 3.0 mg/kg) daily, using a counterbalanced design, and locomotor activity measured immediately post-injection for 60 min.

#### Statistical Analysis

*Human*: Baseline questionnaire data were analyzed using one-way ANOVA with genotype and drug group as a between-subject factors. Mood measurements were analyzed using repeated measures ANOVA with time (pre- vs. post-drug) as within-subject and genotype and drug as between-subject factors. CRf dependent variables were analyzed using mixed ANOVAs with stimulus (CS+ vs. CS−) as within, and drug and genotype as between-subject factors.

*Mouse*: Acquisition of Pavlovian conditioning was assessed by calculating the percentage of nose pokes per minute into the food magazine during the CS presentation. These data were analyzed using a 3-factor mixed ANOVA, with genotype as a between-subject, and session and CS type as within-subject factors. Effects of methylphenidate and genotype on CRf were analyzed using a mixed ANOVA in which the factors dose and nosepoke aperture (CS+ or CS−) were within-subject and genotype between-subject measures.

Methylphenidate effects on locomotor activation (distance travelled) were assessed using a 2-factor mixed ANOVA, with genotype as a between-subject and drug dose as a within-subject factor. *Post hoc* paired-samples *t*-tests compared the test dose to vehicle.

## Results

### Human Studies

#### Group Characteristics and Baseline Measurements

Table 2 shows group characteristics, indicating no difference in age, verbal IQ, or other characetristics.

**Table 2:** **Participants characteristics given for each of the four groups (genotype protective (P) or risk (R) and placebo or methylphenidate treatment)**.

Variables	P Placebo *N* = 12	P Methylphenidate *N* = 11	R Placebo *N* = 14	R Methylphenidate *N* = 12	Statistics univariate analysis *F*s^(1,45)^
Age	20.7 ± 0.9	20.3 ± 0.5	20.7 ± 1.9	21.2 ± 1.0	*F*s < 0.271, *P*s > 0.607
NART (verbal IQ)	102.7 ± 2.9	105.9 ± 3.5	107.1 ± 3.0	105.3 ± 2.5	*F*s < 0.705, *P*s > 0.406
AUQ score	33.7 ± 5.2	36.3 ± 10.8	36.1 ± 6.3	37.3 ± 6.6	*F*s < 0.067, *P*s > 0.797
DUQ	1.0 ± 0.4	0.64 ± 0.3	1.3 ± 0.3	1.7 ± 0.4	*F*s < 3.480, *P*s > 0.069
Cigarettes per day	1.0 ± 0.9	0.68 ± 0.4	1.4 ± 0.9	2.7 ± 1.7	*F*s < 1.139, *P*s > 0.291
Trait anxiety	36.1 ± 2.3	40.9 ± 3.4	36.0 ± 2.0	36.7 ± 2.3	*F*s < 1.254, *P*s > 0.269
State anxiety	31.3 ± 2.1	38.9 ± 2.7	29.0 ± 1.6	30.2 ± 2.6	*F*s < 3.483, *P*s > 0.0697

*No significant main group effects or between group interactions were found; NART, National Adult Reading Test; AUQ, Alcohol Use Questionnaire; DUQ, Drug Use Questionnaire*.

Pavlovian conditioning led to an increase in expectancy of reward in the presence of CS+ vs. CS− (data not shown; main stimulus effect: *F*_1,41_ = 340.8; *p* < 0.001), which increased over trials (block by stimulus interaction (*F*_4, 164_ = 5.573; *p* < 0.001)), showing progression of learning. All participants already showed learning in block 1 of trials. No genotype effects, or interactions involving genotype reached significance (*F* values < 2.345; *p* > 0.1).

#### Methylphenidate Mood Effects

**Figure 2:**
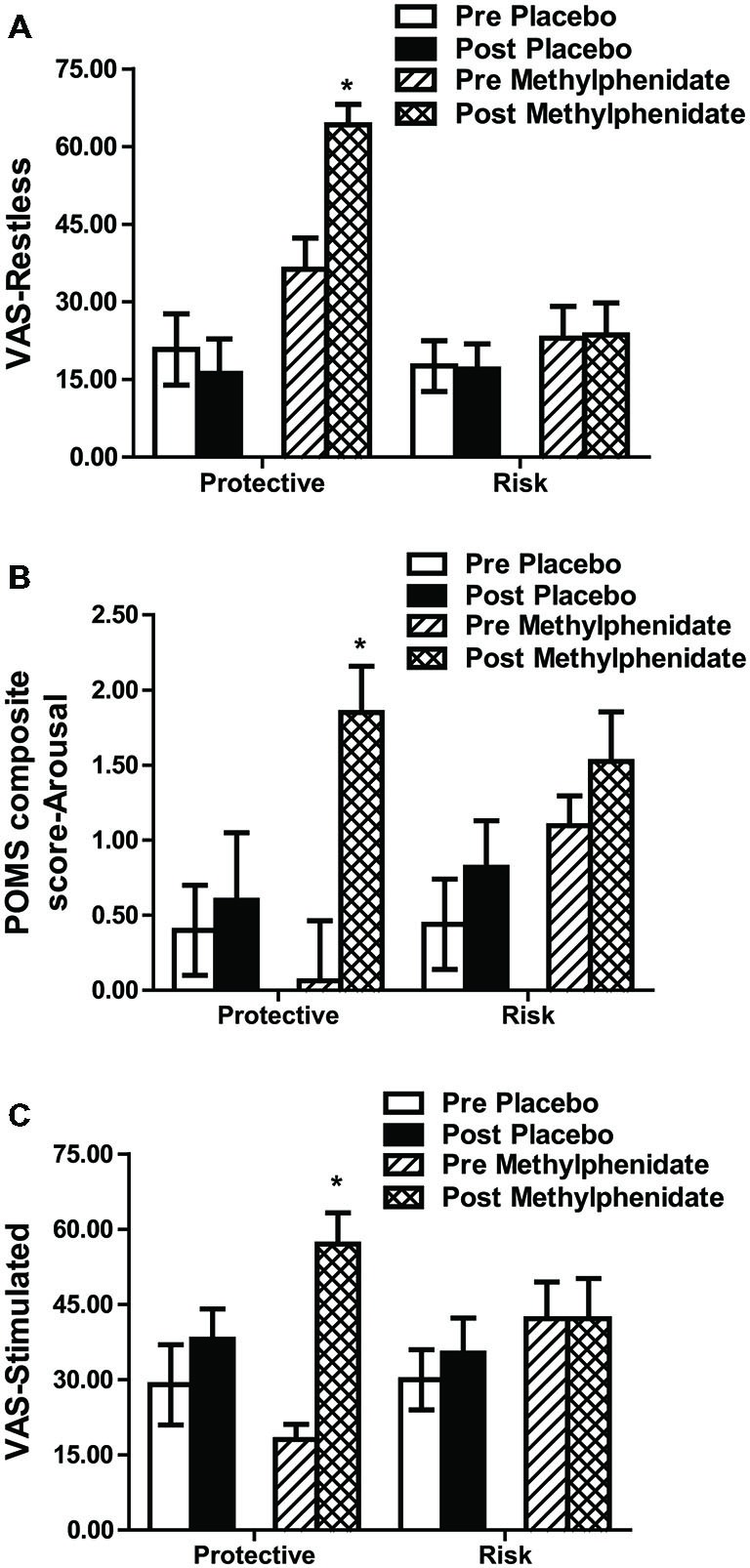
**Subjective ratings in Visual Analog Scale (VAS) restless (A), in the composite score “Arousal” from the Profile of Mood States (POMS; B) and in Visual Analog Scale stimulated (C).** Data (mean ± SEM) are given for risk and protective genotypes and for pre and post methylphenidate or placebo treatments. A significant drug by genotype by time interaction was attributable to an increase in the ratings under methylphenidate in comparison to placebo in the protective, but not in the risk genotype. *p < 0.05 compared to pre drug (*post hoc* paired *t*-tests).

Methylphenidate increased restlessness, as measured by VAS, and arousal, as measured by the Profile of Mood States (POMS), in all participants (time by drug interaction, *F*_1,45_ = 7.334, *p* = 0.010 and *F*_1,45_ = 8.167, *p* = 0.006 respectively); these methylphenidate effects were greater in the protective vs. the risk genotype (genotype by drug by time interaction, “restless” *F*_1,45_ = 6.335, *p* = 0.015 (Figure 2A) and “arousal” *F*_1,45_ = 7.407, *p* = 0.009 (Figure 2B), respectively). VAS measures of feeling “stimulated” showed also larger increases following methylphenidate in the protective vs. the risk genotype (genotype by drug by time interaction (*F*_1,45_ = 7.087, *p* = 0.011; Figure 2C). No effects on measures for “positive mood” (composite factor in POMS) or VAS scales “alert”, “irritable”, “contended”, “anxious”, “lightheaded” and “relaxed” were found (data not shown).

#### Methylphenidate-Potentiated Conditioned Reinforcement

**Figure 3:**
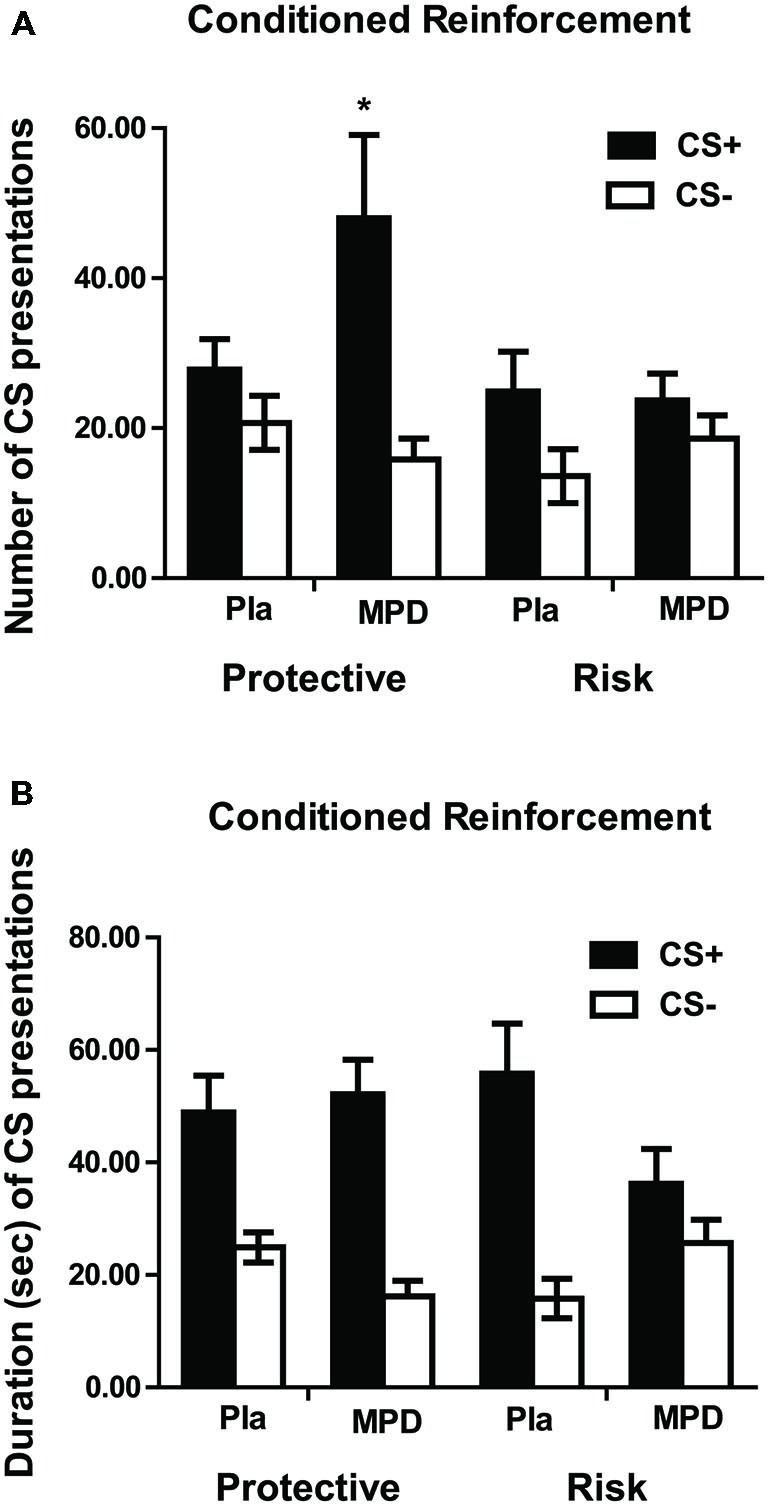
**Number of CS presentations (A) and duration of CS presentations (B) for the CS+ and CS− in the 2 min of CRf measurement.** Data (mean ± SEM) are given for risk and protective genotype and for methylphenidate (MPD) and placebo (Pla) treatments. A significant drug by genotype by stimulus interaction was found for the number of CS presentations due to an increase in the number of CS+ presentations under methylphenidate in the protective but not in the risk genotype. A significant drug by genotype by stimulus interaction was found also for the duration of CS presentations, indicating a decrease in duration of CS+ presentation under methylphenidate, in comparison to placebo, in the risk but not in the protective genotype.

Following Pavlovian pairings of visual stimuli with monetary reward, subjects responded to earn access to the stimuli. The number of CS presentations obtained (*F*_1,45_ = 17.69; *p* < 0.001; Figure 3A) and the total duration of CS presentations (*F*_1,45_ = 47.7; *p* < 0.001; Figure 3B) were greater during CS+ than CS− trials, indicating that the CS+ had acquired (conditioned) reinforcing properties, but there were no genotype differences. Methylphenidate increased the number of CS+ presentations in the protective, but not in risk genotypes (Figure 3A). A significant 3-way (stimulus by drug by genotype) interaction (*F*_1,45_ = 5.597; *p* = 0.022) was explained by an increase in the number of earned CS+ presentations following methylphenidate vs. placebo treatment in the protective (*F*_1,21_ = 4.657, *p* = 0.043), but not the risk genotype (*F*_1,24_ = 0.781, *p* = 0.386). For CS presentation duration, a significant 3-way interaction (*F*_1,45_ = 6.77; *p* = 0.013) was attributable to a drug by stimulus interaction in the risk (*F*_1,24_ = 6.19; *p* = 0.020), but not protective (*F*_1,21_ = 1.33; *p* = 0.26) genotype, with methylphenidate *decreasing* the duration of CS+ presentations in the risk genotype (Figure 3B).

In the protective genotype group, three participants were homozygous for the protective genotype but heterozygous for the two risk alleles. We therefore carried out an additional statistical analysis using only those individuals in the protective genotype group who were homozygous in not possessing the risk variant. The statistically significant three way interaction for number of CS presentations and total duration of CS presentations remained significant (*F*_1,42_ = 4.76; *p* = 0.035 and *F*_1,42_ = 5.68; *p* = 0.022, respectively).

To examine whether the methylphenidate-induced changes in mood varied systematically with the CRf performance (duration of stimulus presentation and number of stimulus presentations), *post hoc* bivariate Pearson correlations were performed between mood ratings and CRf performance, separately for the methylphenidate and the placebo groups. For this purpose baseline VAS ratings “stimulated” and “restless” as well as POMS “arousal” ratings were subtracted from post-drug ratings; duration and number of CS− presentations during CRf, were subtracted from the respective values of CS+.

As expected, significant correlations were found only for the methylphenidate group. Enhanced feelings of “stimulated” were correlated with the number (*r* = 0.463, *p* = 0.026) and the duration (*r* = 0.602, *p* = 0.002) of CS+ over CS− presentations. Duration of presentation of CS+ over CS− increased also with increased feelings of “restless” (*r* = 0.480, *p* = 0.02).

### Mouse

#### Methylphenidate-Potentiated Conditioned Reinforcement

**Figure 4:**
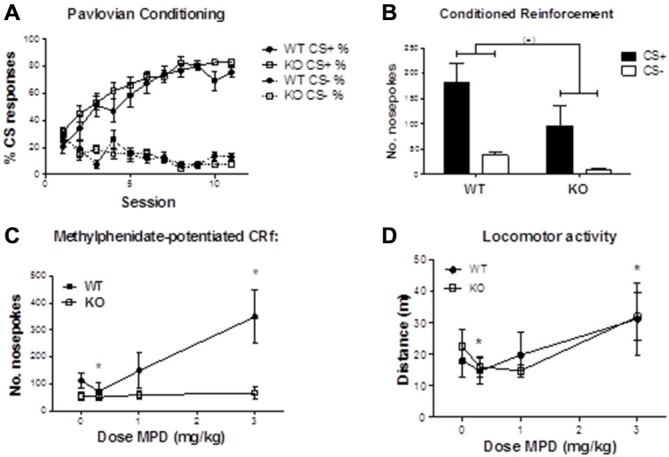
**(A)** Percentage of CS presentations resulting in entry to the food magazine following presentations of CS+ or CS−, over successive training sessions. There were no statistically significant differences in the rate at which WT and α2^−/−^ mice acquired the association between CS+ and food delivery (nosepoke by genotype interaction: *F*_(1,22)_ = 1.322, *p* = 0.263). **(B)** Numbers of head entries into nose-poke detectors resulting in 1-s presentations of either the CS+ or CS−. Although WT mice showed a tendency to make more nosepokes into either CS+ or CS− holes (*F*_(1,22)_ = 4.170, *p* = 0.053), there were no significant interactions between genotype and CS, or in responding for the CS+. **(C)** After methylphenidate treatment, mice showed a dose dependent change in nosepoke responding that differed according to genotype (nosepoke * dose *genotype interaction: *F*_3,51_ = 4.276, *p* < 0.01). This effect was attributable to CS+ responses (dose*genotype interaction: *F*_3,51_ = 4.336, *p* < 0.01) but not the CS− (dose*genotype interaction: *F*_3.51_ = 1.015, *p* = 0.394). The dose dependent effect of responding on the CS+ was evident in WT (*F*_3,30_ = 7.286, *p* < 0.01) but not in the α2^−/−^ mice (main effect of dose: *F*_3,21_ = 0.127, *p* = 0.943). * indicates significant differences from the vehicle condition in WT mice. **(D)** Methylphenidate increased locomotor activity in a dose-dependent manner (*F*_3,66_ = 7.825, *p* < 0.001), independently of genotype. * indicates significant differences from the vehicle condition. *MPD* (Methylphenidate).

*Pavlovian conditioning*. Figure 4A illustrates that both α2^−/−^ and WT mice approached the food receptacle equally following onset of the CS+ (main effect of session: *F*_10,220_ = 32.079, *p* < 0.001; session by genotype interaction: *F*_10,220_ = 0.731, *p* = 0.695), confirming that constitutive α2 deletion has little effect on simple discriminative Pavlovian conditioning (Dixon et al., 2010).

*Conditioned Reinforcement*. On the initial test to verify CRf, mice of both genotypes readily and equally learned to nose-poke to preferentially obtain access to the reward-associated visual cue (Figure 4B; main effect of nose-poke, *F*_1,22_ = 19.604, *p* < 0.001; nosepoke by genotype interaction, *F*_1,22_ = 1.322, *p* = 0.263), indicating that the CS+ had acquired conditioned reinforcing properties. WT mice tended to nose-poke at higher rates (main effect of genotype: *F*_1,22_ = 4.170, *p* = 0.053). One WT and four α2^−/−^ mice failed to meet criterion, and were excluded from subsequent drug testing.

*Methylphenidate-potentiated CRf*. Under methylphenidate, mice showed a dose-dependent change in discriminated nose-poke responding that differed according to genotype (Figure 4C; nose-poke by dose by genotype interaction: *F*_3,51_ = 4.276, *p* < 0.01). The genotype by dose interaction was present for responses reinforced by CS+ (*F*_3,51_ = 4.336, *p* < 0.01) but not for CS− reinforced responses (*F*_3.51_ = 1.015, *p* = 0.394). The dose-dependent effect on responding for the CS+ was evident in WT mice (main effect of dose: *F*_3,30_ = 7.286, *p* < 0.01), whilst methylphenidate had no effect in α2^−/−^ mice (*F*_3,21_ = 0.127, *p* = 0.943). In the WT mice, paired sample *t*-tests comparing methylphenidate to vehicle revealed a significant decrease in CS+ responses at 0.3 mg/kg (*t*_10_ = 3.862, *p* < 0.01) and a significant increase at 3 mg/kg methylphenidate (*t*_10_ = −3.047, *p* < 0.05).

##### Methylphenidate Effects On Locomotor Activity

Figure 4D illustrates that methylphenidate increased locomotor activity in a dose-dependent manner (main effect of dose *F*_3,66_ = 7.825, *p* < 0.001), but that there were no genotype differences in locomotor activity (*F*_1,22_ = 0.002, *p* = 0.963), or drug by genotype interactions (*F*_3,66_ = 0.573, *p* = 0.635).

## Discussion

We and others have previously reported an association between variants of the GABRA2 gene, encoding the α2 subunit of GABA_A_ receptors, and human cocaine addiction (Dixon et al., 2010; Enoch et al., 2010). Since deleting α2-GABA_A_ receptors in mice abolishes cocaine’s ability to facilitate responding for a conditioned reinforcer, we suggested that these findings might provide an approach to understanding the association between GABRA2 variants and human cocaine addiction (Dixon et al., 2010). In the current study, we developed a novel measure of CRf in humans, based upon the mouse task, which allowed us to demonstrate for the first time that a psychostimulant facilitates responding for CRf in non-addicted humans. Individuals carrying risk and protective variants of the GABRA2 gene did not differ in their performance on our task, indicating no effects of the gene variants on incentive learning, consistent with our mouse studies showing no effects of α2 deletion on CRf (Dixon et al., 2010). However, the ability of the psychostimulant, methylphenidate, to facilitate human CRf depended on genetic makeup; while subjects carrying the addiction-protective genotype showed methylphenidate-facilitated CRf, those carrying risk alleles did not. These effects were paralleled in mice; whereas at low dose methylphenidate mildly reduced CRf responding in WT mice, at higher doses it markedly facilitated CRf responding in WT but not α2^−/−^ mice. The reduction of responding for a conditioned reinforcer by 0.3 mg/kg methylphenidate was unexpected, but such a low dose does not appear to have been tested previously. However, facilitation of CRf by methylphenidate in WT mice has been previously reported (Browne et al., 2014). Furthermore, the lack of facilitation of CRf by methylphenidate in the α2^−/−^ mice resembles that previously reported for cocaine (Dixon et al., 2010). Our mouse data also indicate that the ability of methylphenidate to facilitate responding for the conditioned reinforcer was not simply a consequence of its motor stimulant properties since the locomotor stimulant effects of methylphenidate did not differ between genotypes.

A more likely account is that, in the human protective genotype group and in WT mice, methylphenidate either increased the *value* of the conditioned reinforcer, or facilitated behavior elicited specifically by reward-associated cues, thus facilitating responding to obtain presentations of the CS+. Interestingly, in the risk group the value of the conditioned reinforcer seemed to decrease under methylphenidate, inasmuch as the CS+ presentation duration was decreased.

In addition to the altered methylphenidate effect on CRf in the GABRA2 variants, we found higher subjective ratings of methylphenidate-induced stimulation, restlessness and arousal in individuals carrying the protective genotype compared to those subjects expressing the risk genotype (Figure 2). *Post hoc* correlations suggested that feeling “stimulated” under methylphenidate was related to the number and duration of CS+ presentations under the drug. Since the subjective experience of methylphenidate stimulant effects (“high”) paralleled its ability to facilitate CRf performance, the feeling of “stimulated” may in part reflect a subjective experience of a heightened motivational state.

The predominant difference between the two genotypes groups in our study is the presence or absence of two “risk” SNPs. SNAP analysis of data from the 1000 Genomes Project (Johnson et al., 2008) shows our risk SNP rs279871 to be in 100% linkage disequilibrium with rs279858, while the other, rs279845, is in 92.9% LD with rs279858. rs279858 is the common risk SNP recognized across multiple studies of addicted populations; it has been associated with alcohol (Covault et al., 2004; Lappalainen et al., 2005; Fehr et al., 2006) and cocaine abuse (Enoch et al., 2010), as well as childhood conduct disorder (Dick et al., 2006) and impulsivity during reward anticipation (Villafuerte et al., 2012). Thus variations in this genomic area may alone be driving our genotype differences. It may thus be relevant that some (Pierucci-Lagha et al., 2005; Roh et al., 2011) but not all (Arias et al., 2014) previous studies have found decreased effects of another drug of abuse, alcohol, in measures of positive affect such as stimulation, vigour and happiness, in carriers of the C-allele of rs279858.

However, in order to try to increase our effect size within the “risk+” or “risk−” groups we enriched them with the absence (in the risk+ group) or presence (in the risk− group) of the putative protective SNP or haplotype identified in the previous study (Dixon et al., 2010). This counts as a limitation in our study as we cannot be sure whether the effect seen in the present study is driven purely by the recognized risk SNP. Future studies should establish the contributing genetic elements.

Inasmuch as risk genotype individuals have an increased susceptibility for substance abuse or addictions, but reduced effects of methylphenidate on both CRf, and feelings of “restlessness”, “stimulated” and “arousal”, our findings may support accounts that drug taking occurs in order to overcome either an individual’s innate relative insensitivity to reward (Blum et al., 1996; Volkow et al., 1997) or a loss of reward sensitivity as a consequence of “allostatic” adaptations arising from previous drug abuse (Koob and Le Moal, 2008). In keeping with that account, Volkow et al. (1997) reported that cocaine addicts show a blunted cocaine “high” relative to controls. Furthermore, individuals with low dopamine D2 receptor levels and dopamine release show vulnerability to develop addiction either in terms of high impulsive traits (Trifilieff and Martinez, 2014) or decreased reward sensitivity (Blum et al., 2014). Thus, addiction can be understood as being associated with reduced sensitivity to the stimulant subjective effects of abused drugs, including both psychostimulants and alcohol. While in Volkow’s studies, tolerance to cocaine’s subjective effects could have contributed to the findings, our data are also consistent with drug abuse being attributable to low reward-sensitivity (perhaps mediated by dopamine systems; Blum et al., 1996, 2000), independent of drug-experience. In the present study we found some evidence (Table 2; DUQ) that individuals who were not addicts, but carried a genetic risk for addictions, were more likely than those carrying the protective genotype to use drugs recreationally; however the effect was marginal (*p* = 0.067).

On the other hand, our data question the importance of drug sensitization in the abuse of psychostimulants. Though in the present report we did not examine the consequences of alpha 2 knockout on behavioral sensitization in the mouse, it would be remiss not to point out that we have previously found such mice to show attenuated sensitization of cocaine-induced psychomotor activation (Dixon et al., 2010). When taken together with the present human data, these results may thus suggest a dissociation between psychomotor sensitization by cocaine and susceptibility to develop compulsive drug seeking as seen in addicts. This conclusion seemingly challenges Robinson and Berridge’s incentive sensitization theory of addiction (Robinson and Berridge, 1993) but that finds support from others (e.g., Ahmed and Cador, 2006). On the other hand, such a conclusion oversimplifies psychomotor activation both as a measure in the lab (see Robinson and Berridge, 1993) and as a theoretical mechanism in addiction. Indeed, future preclinical studies exploring the involvement of alpha 2 in the effects of sensitization on more direct measures of incentive motivation (e.g., performance on Pavlovian-to-instrumental transfer, CRf or sign-tracking tasks) are needed.

A recent report (Heitzeg et al., 2014) investigated incentive-motivational circuitry in individuals carrying variations in the GABRA2 gene. It is of considerable interest that in that study, adolescents carrying the minor (risk) allele of rs279858 showed a heightened BOLD signal of accumbens during reward anticipation in the Monetary Incentive Delay task, indicating increased accumbal responsiveness to incentive cues. Although the subjects in our study encompassed a broader age range, the majority (*n* = 43) were in the 18–22 range. That risk genotypes result in heightened accumbal response to conditioned incentives is consistent with our earlier suggestion that manipulation of the GABA_A_ α2 subunit in mice alters the “energizing” aspects of reward seeking (Dixon et al., 2014). Since GABA_A_ α2 subunits contribute importantly to GABAergic inhibition of MSNs (Dixon et al., 2010; presumably the major source of the accumbens BOLD signal, as they make up >90% of neurones in ventral striatum), the heightened BOLD response in risk allele carriers would be consistent with reduced α2 subunit-mediated inhibition. The lack of effects of methylphenidate in risk genotype carriers may thus reflect that in these individuals, MSN responsiveness to reward signals is already near maximal.

Thus, in keeping with the Heitzeg et al. (2014) report, a likely location for the alpha2 subunits involved in the present dataset is the ventral striatum, where we have shown that deletion of alpha2 subunits leads to a 30% loss of GABAergic input to MSNs of accumbens core (Dixon et al., 2010). It is well established that ventral striatum core plays a central role in CRf (Everitt and Robbins, 2005), and that facilitation of dopaminergic input to this area lies at the basis of psychostimulant facilitation of CRf (Kelley and Delfs, 1991). However, it would be premature to exclude the importance of GABA_A_ receptors employing alpha2 subunits in amygdala (potentially modulating the transfer of information on conditioned events to striatum) or prefrontal cortex. These are important questions for future study.

Finally, although there is no adequate information available as to whether the risk and protective gene variants result in altered levels of receptors, the parallels between the absence of methylphenidate effects on CRf in the α2^−/−^ mice, and humans carrying the risk allele, suggest the effects of the risk genetic variation may resemble that of the deletion of the GABRA2 gene in α2^−/−^ mice. Thus, the absence of methylphenidate-facilitation of CRf responding in humans carrying risk SNPs for drug abuse may reflect low expression levels of α2. Some support for this interpretation comes from a recent report (Lieberman et al., 2015) that GABRA2 mRNA in neural cell cultures derived from rs279858*C allele (risk allele) carriers is lower. If our interpretation is correct, neurotransmission through α2-GABA_A_Rs may be protective against the development of addictive behaviors. Enhancement of such transmission using drugs selective for α2-GABA_A_Rs might be a novel approach to addiction treatments. The availability of homologous human and mouse measures of responding for conditioned incentives should facilitate the testing of that prediction.

## Conflict of Interest Statement

The authors declare that the research was conducted in the absence of any commercial or financial relationships that could be construed as a potential conflict of interest.

## Footnotes

^1^www.kbioscience.co.uk
